# Hspb1 and Lgals3 in spinal neurons are closely associated with autophagy following excitotoxicity based on machine learning algorithms

**DOI:** 10.1371/journal.pone.0303235

**Published:** 2024-05-10

**Authors:** Lei Yan, Zihao Li, Chuanbo Li, Jingyu Chen, Xun Zhou, Jiaming Cui, Peng Liu, Chong Shen, Chu Chen, Hongxiang Hong, Guanhua Xu, Zhiming Cui

**Affiliations:** The First People’s Hospital of Nantong, Research Institute for Spine and Spinal Cord Disease of Nantong University, The Second Affiliated Hospital of Nantong University, Nantong, China; The First Hospital of Jilin University, CHINA

## Abstract

Excitotoxicity represents the primary cause of neuronal death following spinal cord injury (SCI). While autophagy plays a critical and intricate role in SCI, the specific mechanism underlying the relationship between excitotoxicity and autophagy in SCI has been largely overlooked. In this study, we isolated primary spinal cord neurons from neonatal rats and induced excitotoxic neuronal injury by high concentrations of glutamic acid, mimicking an excitotoxic injury model. Subsequently, we performed transcriptome sequencing. Leveraging machine learning algorithms, including weighted correlation network analysis (WGCNA), random forest analysis (RF), and least absolute shrinkage and selection operator analysis (LASSO), we conducted a comprehensive investigation into key genes associated with spinal cord neuron injury. We also utilized protein-protein interaction network (PPI) analysis to identify pivotal proteins regulating key gene expression and analyzed key genes from public datasets (GSE2599, GSE20907, GSE45006, and GSE174549). Our findings revealed that six genes—Anxa2, S100a10, Ccng1, Timp1, Hspb1, and Lgals3—were significantly upregulated not only in vitro in neurons subjected to excitotoxic injury but also in rats with subacute SCI. Furthermore, Hspb1 and Lgals3 were closely linked to neuronal autophagy induced by excitotoxicity. Our findings contribute to a better understanding of excitotoxicity and autophagy, offering potential targets and a theoretical foundation for SCI diagnosis and treatment.

## Introduction

Glutamate is a well-known excitatory neurotransmitter in the central nervous system (CNS). It has been long recognized that following spinal cord injury (SCI), elevated levels of glutamate can result in excessive and sustained activation of glutamate receptors, ultimately leading to cell death, known as excitotoxicity [1,2]. The mechanism of excitotoxicity is primarily associated with the dysregulation of glutamate receptors and intracellular/extracellular ion concentrations across the cell membrane [3]. Excitotoxicity has been widely acknowledged as a key factor contributing to neurological deficits and neuronal demise post-SCI [4–11]. Furthermore, excitotoxicity induced by high levels of glutamate is implicated in neuronal cell death following acute and chronic injuries to the central nervous system, such as stroke and brain trauma [12]. Researchers have utilized elevated levels of glutamate to induce neuronal damage in primary neurons. For instance, in 2009, Kim observed the activation of autophagy during glutamate-induced cell death in mouse hippocampal HT22 cells [13]. Additionally, Chen demonstrated an increase in the autophagy levels of HT22 hippocampal neurons following glutamate stimulation to induce nerve injury [14]. Moreover, Vongthip employed glutamate to induce neurotoxicity and cell death [15]. Despite these studies shedding light on the potential interplay between excitotoxicity and autophagy, the specific mechanistic relationship between excitotoxicity and autophagy has been largely overlooked.

SCI results from direct or indirect external factors causing damage to the spinal cord, leading to a range of motor, sensory, and sphincter dysfunctions, dystonia, and pathological alterations in the corresponding segments [16]. The pathophysiological processes of SCI at the cellular level encompass a myriad of injury responses, including excitotoxicity, ischemia, oxidative stress, inflammation, and apoptosis [17], ultimately resulting in neuronal autophagy and apoptosis, axonal demyelination, Wallerian degeneration [4], and ultimately extensive and permanent neuronal loss, culminating in sensory, motor, and autonomic nervous dysfunction [18]. SCI remains an incurable condition that has perplexed clinicians for years. The current understanding of the pathological processes of SCI has spurred numerous investigations into potential treatment mechanisms. Among these, the inhibition of neuronal autophagy and apoptosis is a critical aspect of SCI repair and treatment, playing a pivotal role in managing acute and chronic SCI pathogenesis [19]. Therefore, investigating the excitotoxicity mechanisms of neurons post-SCI is crucial for developing neuroprotection strategies and enhancing neurological function recovery following SCI.

Autophagy refers to the process in which cells maintain the internal environment’s homeostasis through self-degradation. In the context of SCI, autophagy plays a crucial and intricate role, garnering significant attention from researchers [20–22]. The role of neuronal autophagy in SCI has long been recognized as dual in nature, capable of exerting both protective and pathological functions [23,24]. Studies have indicated that the upregulation of autophagy markers such as Atg5 and LC3-II, along with the downregulation of P62, is associated with a protective role in eliminating spinal cord and nerve cell damage [25]. Conversely, research has also shown that the inhibition of autophagy, such as with the use of 3-MA, can decrease autophagic activity, reduce neuronal degeneration, and enhance neurological function recovery [26]. The role of autophagy post-SCI is heterogeneous and complex, with potential changes over time [23,24,27]. Early after injury, autophagy may aid in the elimination of damaged cells and the maintenance of energy metabolism, whereas in later stages, it may play a role in axon regeneration and repair [27,28]. Despite the attention given to autophagy in SCI, further research is required to elucidate the mechanisms of excitotoxicity and autophagy in injured neurons.

As a member of the heat shock protein family, Hspb1 has been shown to mitigate stress-induced cellular damage and participate in various cellular processes including apoptosis, redox balance, and the regulation of cytoskeleton dynamics [29]. Haidar’s research has demonstrated that Hspb1 exerts a regulatory influence on autophagy by directly interacting with SQSTM1, and the detrimental impact of Hspb1 mutations on motor neurons has been confirmed in patient studies [30]. Additionally, consensus has been reached on the pivotal role played by small heat shock proteins in the pathological progression of neuronal demise in human neurodegenerative disorders [31]. Belonging to the β-galactoside-binding cytoplasmic lectin family, Lgals3 is involved in the regulation of diverse cellular responses and essential biological functions, encompassing immune response, proliferation, differentiation, migration, and cell growth [32]. In neurodegenerative conditions such as Alzheimer’s disease (AD), Parkinson’s disease (PD), and amyotrophic lateral sclerosis (ALS), Lgals3 has been established as a biomarker in serum, plasma, and/or cerebrospinal fluid (CSF) [33]. Recent studies have revealed the direct participation of Lgals3 in the regulation of autophagy induced by lysosomal stress resulting from SNCA fibrils in dopamine (mDA) neurons within the human midbrain [34]. However, the roles of Hspb1 and Lgals3 in excitotoxicity and autophagy in spinal cord neurons following SCI have been minimally documented.

High-throughput chip sequencing technology has been widely used in many aspects such as basic medical research and disease diagnosis. Through in-depth research on the knowledge of molecular biology and genomics, we can better understand the relationship and the mechanism of molecules in organisms, so as to provide support for the development of new drugs, the development of treatment options, and the search for cellular genetic markers [35]. Bioinformatics is a practical interdisciplinary subject that combines molecular biology and information technology. It carries out comprehensive data analysis and mining on research objects such as the genome, transcriptome, and protein group, so as to discover the potential rules and significance. This research method can not only reveal the mechanism of disease occurrence and development, but also provide a new way for disease diagnosis and treatment [36]. Machine learning is an automatic learning method based on data. It uses a large amount of historical data to train algorithm models and find rules and patterns to predict or classify future data [37]. By machine learning algorithms, we can construct prediction and analysis models in bioinformatics research and mine potential biological processes and mechanisms from a large amount of biological data [37]. In short, due to the continuous expansion of the scale of biological data and the rapid development of machine learning algorithms, the cross-application between bioinformatics and machine learning has formed a unique and important field, which provides effective support for biomedicine.

In this study, a high concentration of glutamate was employed to induce stimulation in spinal cord neurons, simulating excitotoxic damage akin to that observed in neurons following SCI. Utilizing sequenced machine learning algorithms and bioinformatics tools, the genes that are pivotal in neuronal excitotoxicity and their closely associated downstream signaling pathways were delineated. This exploration shed light on the potential pathological mechanisms underpinning excitotoxicity in neurons post-SCI, thereby presenting potential targets and a theoretical framework for the diagnosis and treatment of SCI.

## Materials and methods

### Extraction and culture of primary spinal neurons

One-day-old Sprague-Dawley rats were purchased from the Experimental Animal Center of Nantong University, and primary spinal neurons were extracted according to the following methods: (1) The neonatal rats were decapitated after alcohol disinfection. The spinal cord was separated and taken out completely. The adventitia of the spinal cord was stripped off using a stereoscopic microscope in pre-cooled L-15 buffer (Invitrogen). L-15 was sucked away, and 0.125% trypsin was used to cut the spinal cord into pieces. The spinal cord was digested at 37°C for 30 min and gently shaken evenly every 10 min. (2) Tryptase was sucked out, and F-12 medium (Invitrogen)+10% Fetal Bovine Serum (Invitrogen)+10% Penicillin-Streptomycin (Invitrogen) was added to stop the digestion. The supernatant was removed by centrifugation at 1000 rpm for 3 min. L-15 was added again to wash the precipitate gently, and the supernatant was removed by centrifugation at 1000 rpm for 3 min. (3) L-15 was added and mixed gently, followed by filtration of all the liquids by a 70 μm sieve (Beyotime). The filtered liquids were added gently in a 1:1 ratio to the upper layer of motor neuron separation solution [Nycoprep 1.077 (15%) (axis-shield) + Hibernate-E medium (83%) (Invitrogen)+ Brewer’s B27 (2%) (Steamcell) (immiscible) and centrifuged at 2000 rpm × 15 min. (4) The middle layer at the junction of the liquid surface was sucked out and transferred into a new centrifuge tube. The supernatant was removed by centrifugation at 1000 rpm×3 min. Neuron-specific culture medium [containing 2 mmol/L L-glutamine and 2% concentration of B27 neuron-specific supplement with final concentrations of 100 U/mL and 0.1 mg/mL penicillin and streptomycin, respectively] were added and inoculated in the 6-well plates, the cell density of each well was about 8×10^5^ /ml, for cell culture at 37°C in an incubator containing 5% carbon dioxide. (5) One-half of the medium was replaced 24 h after plating, and then every 2 days. To improve the purity of neurons, Ara-C 0.05 mg/mL was added at 24 h after plating. (6) Model of nerve injury: After three days of cell culture, the experimental group used the culture solution containing 10 μM glutamate to stimulate the cells for 1 h [38,39], and the control group added the same amount of solvent phosphate buffer saline (PBS) used to dissolve the stimulant. The culture was then continued for 16 h in a dedicated medium for glutamate-free neurons.

### High-throughput sequencing of primary spinal neurons

Total RNA was isolated from neonatal Sprague-Dawley rat spinal cord neuron cells by TRIZOL reagent (Invitrogen) in collaboration with Allwegene (https://allwegene.com/), by sequencing using the Illumina high-throughput sequencing platform (HiSeqTM2500/4000). In order to analyze transcriptomics more comprehensively, accurately and reliably, NCBI (http://asia.ensembl.org/index.html) and Ensembl (https://www.ncbi.nlm.nih.gov/genome/) were used as reference genomes. Six samples were sequenced, including three control groups and three glutamate-induced groups. The data returned by the company is the expression matrix after batch effect removal, standardization and correction of intra-group differences.

### Gene set enrichment analysis

**Fig 1:**
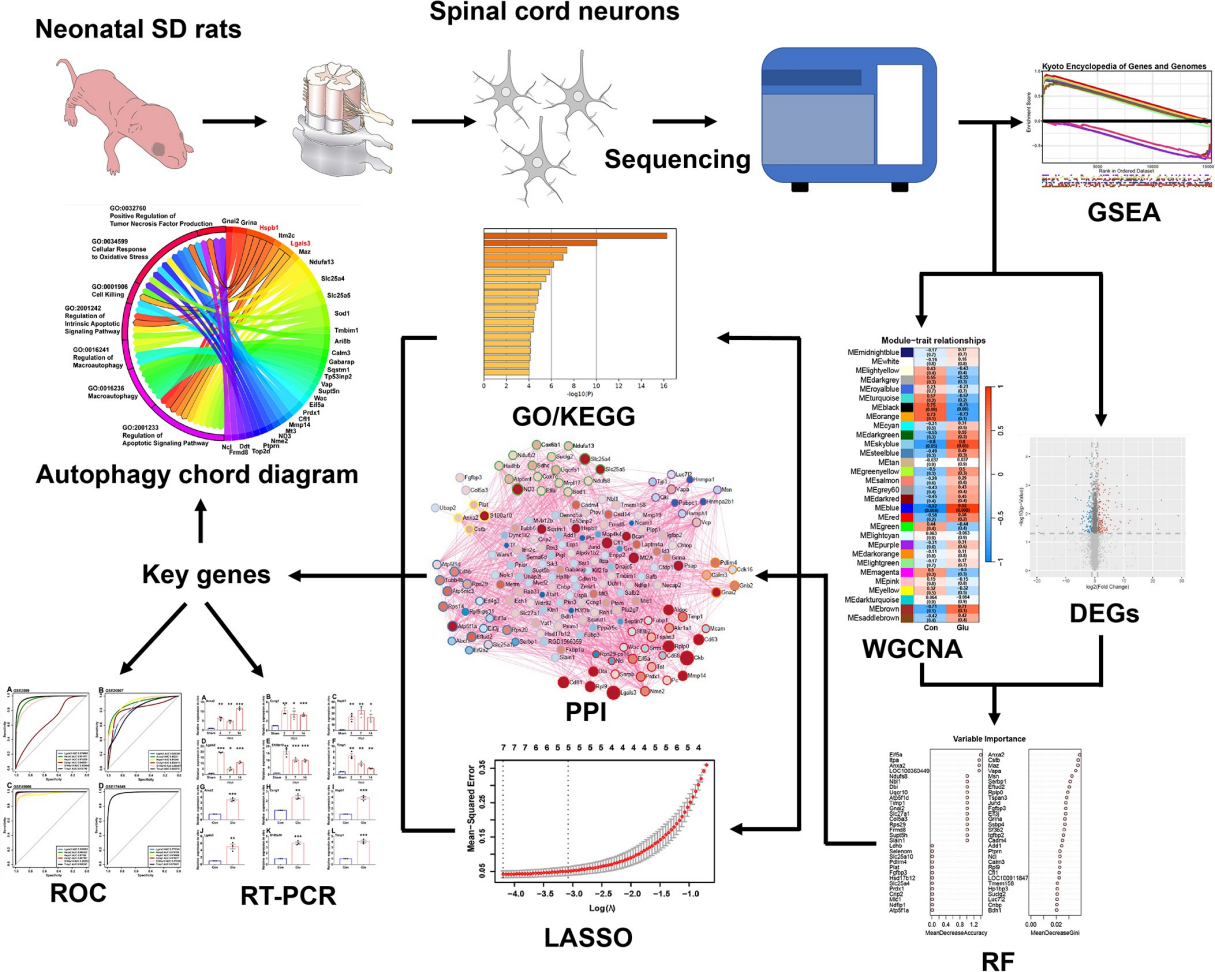
Research roadmap. SD rats, Sprague-Dawley rats; GSEA, Gene Set Enrichment Analysis; WGCNA, weighted correlation network analysis; DEGs, differential expression genes; RF, random forest; GO, Gene Ontology; KEGG, Kyoto Encyclopedia of Genes and Genomes; PPI, protein–protein interaction; LASSO, least absolute shrinkage and selection operator; ROC, Receiver Operating Characteristic; RT-PCR, real-time polymerase chain reaction.

To ensure the accuracy and comparability of the data, the expression matrix obtained from the sequencing company has been pre-processed, including data normalization, removal of batch effects, and transformation of gene expression values. We used R-project (v 3.4.1) for bioinformatics analysis. Because of its concise and intuitive grammar, high customization, unified graphic concept, powerful layer system and rich expansibility, the R package ggplot2 (v 3.4.1; https://cran.r-project.org/web/packages/ggplot2/index.html) was used as the mapping tool for analysis results [40]. We used the gene set enrichment analysis (GSEA) [41] as a calculation method to determine whether a set of apriori-defined genes show statistically significant consistent differences between two biological states by the R package of clusterProfiler [42] (v 4.0.5; https://cran.r-project.org/web/packages/clusterProfiler/index.html). GSEA evaluates the enrichment level of gene sets in the samples by calculating an enrichment score. The significance of gene sets and identification of important signaling pathways and functional features within the gene sets can be determined based on the enrichment score and P-value. The selection criteria for GSEA were set at P < 0.05 with the top ten enrichment scores. The selected species for the gene sets was the rat. The flow chart shows the overall experimental design (Fig 1).

### The weighted correlation network analysis

Because of its statistical analysis based on linear model, efficient processing of large-scale data, flexible and customizable analysis process, multiple test correction and false positive control, and rich visualization tools and results interpretation, the R package Limma (v 3.54.2; http://www.bioconductor.org/packages/release/bioc/html/limma.html) was used to identify differentially expressed genes (DEGs) [43]. The selection of DEGs was carried out by comparing the gene expression levels in spinal cord neurons under control conditions and high-glutamate levels, identifying important genes associated with neuronal excitotoxicity. This approach aids in elucidating the functions and regulatory mechanisms of these gene sets in neuronal excitotoxicity. The screening criteria of DEGs were |logFC| > 1 and P < 0.05.

The weighted correlation network analysis (WGCNA, v 1.6.1; https://cran.r-project.org/web/packages/WGCNA/index.html) is a commonly used machine learning algorithm that serves as a tool [44] for constructing gene co-expression networks and identifying gene clusters or modules. Using WGCNA, a machine learning algorithm, hierarchical clustering methods were applied to analyze the modular structure of the gene co-expression network in spinal cord neurons subjected to excitotoxicity, identifying gene modules with similar expression patterns. Additionally, the algorithm computes the correlations between different gene modules to identify those with similar biological functions or metabolic pathways. There were cutting height = 0.90, Z-score ≥ 5, and stability-related stability correlation P ≤ 0.05 in this study. The connection of nodes (genes) between the two was used to calculate the dataset, and genes with a correlation coefficient < 0.5 were excluded.

### Random forest analysis

Random forest (RF, v 4.7.1.1; https://cran.r-project.org/web/packages/randomforest/index.html) is an integrated algorithm composed of decision trees and one of the commonly used machine learning algorithms. It can be used to classify problems or regression problems [45]. To enhance the accuracy and reliability of the results, we employed RF as a second machine-learning algorithm to rigorously screen the feature gene sets associated with neuronal excitotoxicity. Genes with variable importance measures > 0 were selected for further detailed functional analysis.

### Protein-protein interaction networks analysis

The Kyoto Encyclopedia of Genes and Genomes (KEGG), Gene Ontology (GO) and protein-protein interaction networks (PPI) analysis was performed using the Metascape website (http://www.metascape.org/) [46]. The connectivity (degree) and hub nodes (genes) in PPI [47] were obtained using scale-free property to obtain. In order to help understand the similarities and differences of biological functions between nodes, the Mode algorithm was applied to this network, and GO enrichment analysis was applied to each Mode network, each Mode network being assigned the color of a stroke [48]. For the hub nodes, the size of the shape represented the value of Mode-degree. The results of PPI were imported into Cytoscape software (v 3.9.1; http://www.cytoscape.org/) and further analyzed in combination with the results of DEGs.

### The least absolute shrinkage and selection operator regression analysis

The least absolute shrinkage and selection operator regression analysis (LASSO) [49] (v 4.1.4; https://cran.r-project.org/web/packages/glmnet/index.html) is also one of the commonly used machine learning algorithms, which is characterized by feature (genes) selection and feature importance ranking while fitting the generalized linear model. LASSO reduces the coefficients of some features to zero by determining the appropriate value of regularization parameter λ, thus realizing feature selection. Among the features selected by LASSO, the importance of features can be evaluated by observing the coefficient of each feature. A larger coefficient indicates a stronger correlation with the response variable (excitotoxicity). In this study, we utilized the LASSO regression model as the final machine learning algorithm. By calculating the values of LASSO-Cox coefficients and ranking them, we identified key genes that are involved in neuronal excitotoxicity in spinal cord neurons.

### Receiver operating characteristic curve verification of dataset

Heatmap was produced using the R package ComplexHeatmap (v 2.14.0; https://cran.r-project.org/web/packages/ComplexHeatmap/index.html) and candidate genes expression levels were compared. We downloaded the corresponding expression levels of key genes of GSE2599 [50], GSE20907 [51], GSE45006[52], and GSE174549 [53] from the Comprehensive Gene Expression Database (https://www.ncbi.nlm.nih.gov/geo/) and used the R package PRROC [54] (v1.3.1; https://cran.r-project.org/web/packages/PRROC/index.html) to make AUC-ROC (area under the curve—receiver operating characteristic curve) of key genes in animal level to judge the ability of SCI. There is the basic information of four datasets (Table 1).

**Table 1:** Basic information of four datasets.

GSE ID	GSE2599	GSE20907	GSE45006	GSE174549
**Platform**	GPL85	GPL6247	GPL1355	GPL25947
**Sham vs SCI (Unit: quantity)**	3 vs 3	4 vs 20	4 vs 20	3 vs 3
**Year**	2004	2010	2013	2022
**Gender**	Female	Female	Female	Female
**Weight/Age**	165–200 g	77±10days	250 g	250 g
**Species**	Rattus norvegicus	Rattus norvegicus	Rattus norvegicus	Rattus norvegicus
**Injury site**	Thoracic 8	Thoracic 9	Thoracic 7	Thoracic 8–10
**Damage details**	The T7 and T9 spinal processes were clamped in a spinal frame, and contusions were made by rapidly displacing the cord 1.0 mm (moderate injury).	The animal was positioned and secured into the frame of an NYU Impactor by clamping the T8 and T11 spinous processes, followed by a moderate spinal contusion injury, dropping a 10 g rod from a height of 25 mm using the MASCIS protocol.	Rats underwent a T6-T8 laminectomy and then received a 35 g clip (Walsh) moderate to severe compression injury at T7 for 1 min.	An impactor weighing 10 g was dropped vertically from a height of 50 mm onto the exposed T9 spinal cord surface, causing a contusion injury.

### Establishment of animal SCI model

Healthy adult female Sprague-Dawley rats (8 weeks old, weighing 180–220 g) were purchased from the Animal Center of Nantong University (Nantong, China). All the purchased rats’ feeding and the animal experiments were completed at Nantong Animal Experimental Center, and all the operations aligned with the specifications. Rats underwent general anesthesia (20 ml/kg) by intraperitoneal injection of avertin (2, 2, 2-tribromoethanol, Sigma-Aldrich) in 0.9% saline solution. They were injured by impact compression using a 35 g Walsh clip for 1 min at thoracic level T 7-T 9 and sacrificed (CO_2_ asphyxia) for spinal cord tissue removal 3, 7, and 14 days after SCI. The Sham group only underwent laminectomy with the exposed spinal cord. Six rats were in each group. N = 30. Apart from the Sham group, there were 8 rats in each experimental group, with 2 rats in each experimental group succumbing during the study. All animal procedures were approved by the Animal Experimental Ethics Committee of Nantong University (Protocol number: S20211229-408) and were carried out in strict accordance with the Guidelines for the Care and Use of Laboratory Animals (8th edition, revised in 2010) of the National Institutes of Health.

### Real-time polymerase chain reaction

Extract the spinal cord segment (within 5 mm) at the center of the SCI site in rats. Total RNA was isolated using TRIZOL reagent (No. A33250, Invitrogen) and were reverse transcribed into cDNA according to the manufacturer’s instructions. Add 500 μl of Trizol and mix well. Centrifuge, discard the precipitate, add 200ul chloroform, shake, and allow to stand for 5 minutes. Aft centrifuging, the upper wat phase is sucked, and isoamyl alcohol with the same volume is added, mixed evenly, and allowed to stand for 5 minutes. The supernatant was discarded by centrifugation and added 1 ml of 75% ethanol was gentle oscillation and suspension precipitation. The RNA concentration was determined after drying. The real-time polymerase chain reaction (RT-PCR) was performed using SYBR green dye (Takara) in a thermal cycler under the following parameters: initial denaturation step at 95 °C for 30 min; 40 cycles at 95 °C for 5 s; And 60 °C for 30 s. The total cDNA of all samples was 1 μg during PCR amplification. The PCR results were quantified by 2^-ΔΔCT^. The entire experimental procedure was completed independently for each sample. There are the mRNA primers (Table 2).

**Table 2:** Key genes primers used in this study.

Gene	Name	Forward primer	Reverse primer
Gapdh	Glyceraldehyde-3-Phosphate Dehydrogenase	ACAGCAACAGGGTGGTGGAC	TTTGAGGGTGCAGCGAACTT
Anxa2	Annexin A2	GTGTGCCACCTCCAGAAAGT	GGAGTCATACAGCCGGTCAG
Ccng1	Cyclin G1	TCCCGCTGGCAACTGATTT	CAAATGGCAAGGTCTCCCGA
Hspb1	Heat Shock Protein Family B (Small) Member 1	TGAGGAGCTCACAGTTAAGACC	GGTGAAGCACCGAGAGATGT
Lgals3	Galectin 3	AGGCTCCTCCTAGTGCCTAT	CCTCCAGGCAAGGGCATATC
Timp1	TIMP Metallopeptidase Inhibitor 1	AATGCCACAGGTTTCCGGTT	GGGCTCAGATTATGCCAGGG
S100a10	S100 Calcium Binding Protein A10	AAAATCAAAAAGACCCTCTGGC	TAGGGAAAAGAAGCTCTGGAAG

### Chord diagram of autophagy

Chord diagram is an innovative drawing tool, which is used to visualize the affiliation between autophagy-related pathways and key autophagy genes [55]. All autophagy pathways were extracted in the GO enrichment analysis of candidate genes and encapsulated using the R package Circlize [55] (v 0.4.15; https://cran.r-project.org/web/packages/circlize/index.html) to draw the chord diagram, determining the key genes associated with it. The drawing step was carried out according to the instructions of the Circlize.

### Enzyme-linked immunosorbent assay

The expression levels of Beclin-1 (No. E1025m, EIAab), Hspb1 (No. E0693m, EIAab), and Lgals3 (No. E0497r, EIAab) in primary spinal cord neurons were determined by ELISA kit. Store the sample at -80 °C before measurement. After diluting the sample with PBS, 100 μl of each well was added to the well plate coated with antibody in the kit, and negative control was established, and incubated at 37 °C for 1.5 h. PBS was washed for 3 min × 3; 50 μl of enzyme-labeled secondary antibody diluted with PBS was incubated at 37 °C for 1 h, and PBS was washed for 3 min × 3; Add 50 μl of substrate solution to each well and left at room temperature to avoid light for 10 min; After color development, add 50 μl termination solution to each well to terminate the reaction. The OD value of 450 nm was read by an enzyme-labeled instrument to determine the antibody level of the sample. The optical density at 450 nm was calculated by subtracting the background value, and the standard curve was drawn.

### Data analysis

GraphPad Prism 9.0 (GraphPad Software Inc.) was used for all statistical analyses. The normality of the quantitative variable was tested and if the data were normally distributed, it was expressed as the mean and standard error (SE). Data were statistically analyzed by unpaired student T-test. If the data were not normally distributed, the quantitative variables were expressed as the median of the range and were compared between groups using Mann Whitney Wilcoxon’s test, respectively. Pearson correlation coefficient test was used in correlation analysis. One-way ANOVA was used to evaluate the significance of the differences between groups in the data. All significance levels were set to P < 0.05.

## Results

### High-throughput sequencing and GSEA of primary spinal neurons

Glutamate-induced the primary spinal cord neurons to simulate the neuron injury model after SCI has been applied in some studies [16,17]. We extracted primary spinal cord neurons from newborn rats, and after being stimulated by high-concentration of glutamate, it was found by CCK8 kit that when the cells were stimulated by 10 μM glutamate culture solution for 1h, the neuron damage effect reached the threshold, and the model establishment effect was the best (S1 Fig).

**Fig 2:**
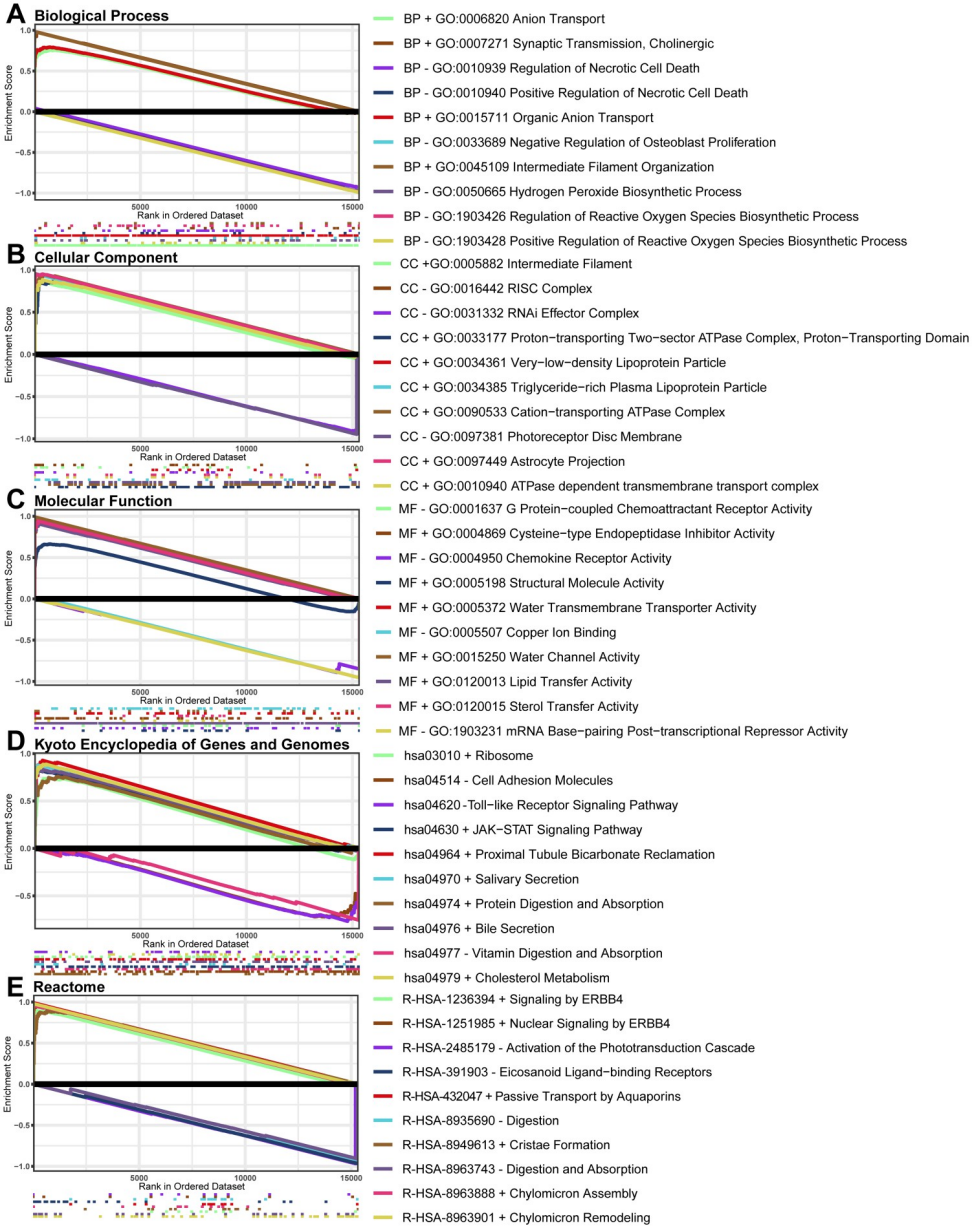
GSEA of primary spinal neurons. **A** The Biological Process results of GSEA. **B** The Cellular Component results of GSEA. **C** The Molecular Function results of GSEA. **D** The Kyoto Encyclopedia of Genes and Genomes results of GSEA. **E** The Reactome results of GSEA. **+** indicated that this gene set was enriched at the top with an up-regulation trend,—indicated that this gene set was enriched at the bottom with a downregulation trend. GSEA, Gene Set Enrichment Analysis.

After sequencing, we found that 15345 genes were analyzed by GSEA, with the results of enrichment analysis in the top 10 by P values (Fig 2). The GSEA analysis of this study includes Biological Process (BP), Cellular Component (CC), Molecular Function (MF), KEGG and Reactome. BP describes the main biological functions of gene products. CC describes the position where gene products play a role. MF describes the molecular tasks of gene products, such as transcription factors or carrier proteins. KEGG and Reactome describe the signal pathways of gene products. Combining these five analysis methods helps understand the biochemical function of gene products in neuron injury models. In the BP results (Fig 2A and S1 Table), *positive regulation of phospholipid transport*, *Golgi to lysosome transport*, and *chaperone-mediated autophagy*, such as these gene sets which are closely related to the formation and occurrence of autophagy are obviously upregulated. Additionally, *positive regulation of reactive oxygen species biosynthetic process*, *hydrogen peroxide biosynthetic process*, *positive regulation of necrotic cell death*, *regulation of necrotic cell death*, and *regulation of reactive oxygen species biosynthetic process*, *cytokine production involved in inflammatory response* was a downregulated trend, suggesting severe resistance to oxidative reaction and neuroinflammatory reaction in spinal neurons. In the CC results (Fig 2B and S2 Table), *cytoplasmic side of lysosomal membrane*, *protein-lipid complex*, *intrinsic component of mitochondrial membrane*, and *lytic vacuole*, etc., these were upregulated trends, suggesting a correlation with mitochondrial autophagy and lysosomal stress. In the MF results (Fig 2C and S3 Table), *antioxidant activity*, *lipid transfer activity*, and *lipoprotein particle binding*, etc., these are upregulation trends, indicating that it is related to cell stimulation and damage. In the KEGG (Fig 2D and S4 Table) and Reactome results (Fig 2E and S5 Table), *JAK-STAT signaling pathway*, *Chaperone Mediated Autophagy* and *Autophagy*, these autophagy-related gene sets are obviously upregulated. Generally speaking, spinal cord neurons in this study developed inflammation, oxidative stress, autophagy, and cell death under glutamate-induced injury.

### Screening of candidate genes

**Fig 3:**
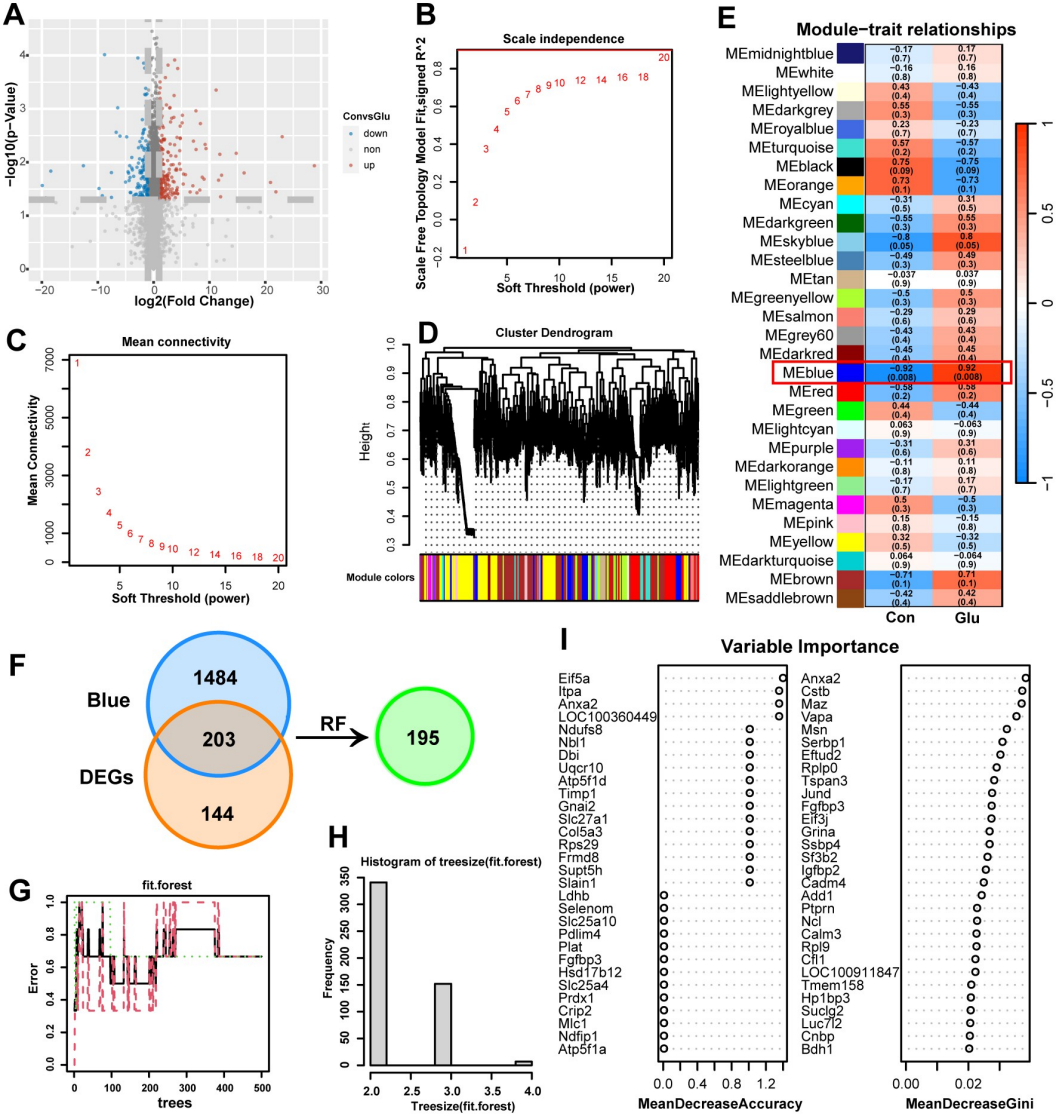
WGCNA and RF of DEGs. **A** Volcano plot of spinal cord neuron control group compared with glutamate stimulated group. **B** Scale independence. **C** Mean connectivity. The network topology analysis for adjacency matrix with different soft threshold power. Red numbers in the boxes indicate the soft thresholding power corresponding to the correlation coefficient square value (y-axis). **D** Consensus module dendrogram was produced by 7340 DEGs with a variation coefficient of expression > 0.1, based on the criteria of correlation coefficient square of eigengenes above 0.90, soft threshold power of 20, the number of genes > 100, and cut height = 0.90. **E** Module-trait relationships. Each row corresponds to a module trait gene, and each column corresponds to a trait. Red indicated a positive correlation between modular trait genes and traits, and blue indicated a negative correlation. Each cell contains the correlation coefficient Rho and the P-value in parentheses. **F** Venn diagram of candidate genes was screened out in the cross set of Blue module and DEGs. **G** Number of optimal decision trees to include in the random forest (ntree = 400). **H** The specific distribution of each category of 203 genes input in a two-dimensional case. **I** The characteristic variables in random forest are arranged in descending order of VI (variable importance). WGCNA, weighted correlation network analysis; RF, random forest; DEGs, differential expression genes; RF, random forest.

347 DEGs, including 145 downregulated genes and 202 upregulated genes, were detected in the sequencing results (Fig 3A). 15345 genes in the results were used as input data for network construction. According to the prerequisite of the approximate scale-free topology processed by WGCNA, the soft threshold power of the adjacency matrix is 20, and the standard that the square of the intrinsic gene correlation coefficient is greater than 0.90 is used as the standard for module identification (Fig 3B). If the gene module is constructed according to the approximate scale-free topology standard, the average connectivity will be 1 (Fig 3C). After the soft threshold was determined, the expression matrix of the differential genes was converted into an adjacency matrix, a topology matrix, and a dissimilarity matrix between genes. On this basis, the hierarchical clustering method was used for gene clustering, and the dynamic cutting algorithm was used for module identification of the system clustering tree (Fig 3D). Thirty different co-expression modules were obtained and expressed in different colors. These modules are associated with the grouping characteristics and the modules that look for continuous correlations (Fig 3E). From the results, it could be seen that the correlation coefficient with SCI in the Blue module was the highest (Rho 3 = 0.92) and the P value was the smallest (P = 0.008), suggesting that the genes in this module were the most relevant to the neuronal damage after glutamate stimulation. The Blue module contains 1687 genes. Then, the intersection of the Blue module and DEGs was continued to extract 203 genes (Fig 3F). Then the characteristic variable (i.e. gene) which is highly related to the neuronal damage after glutamate stimulation is selected through RF. We calculated the optimal number of decision trees (ntree = 400) included in the random forest (Fig 3G), and showed the specific distribution of each type of the input 203 genes in two dimensions (Fig 3H). The characteristic variables in the random forest were sorted in descending order of VI (Variable Importance), and we screened out a total of 195 candidate genes (Fig 3I).

### Screening of key genes from the candidate genes

**Fig 4:**
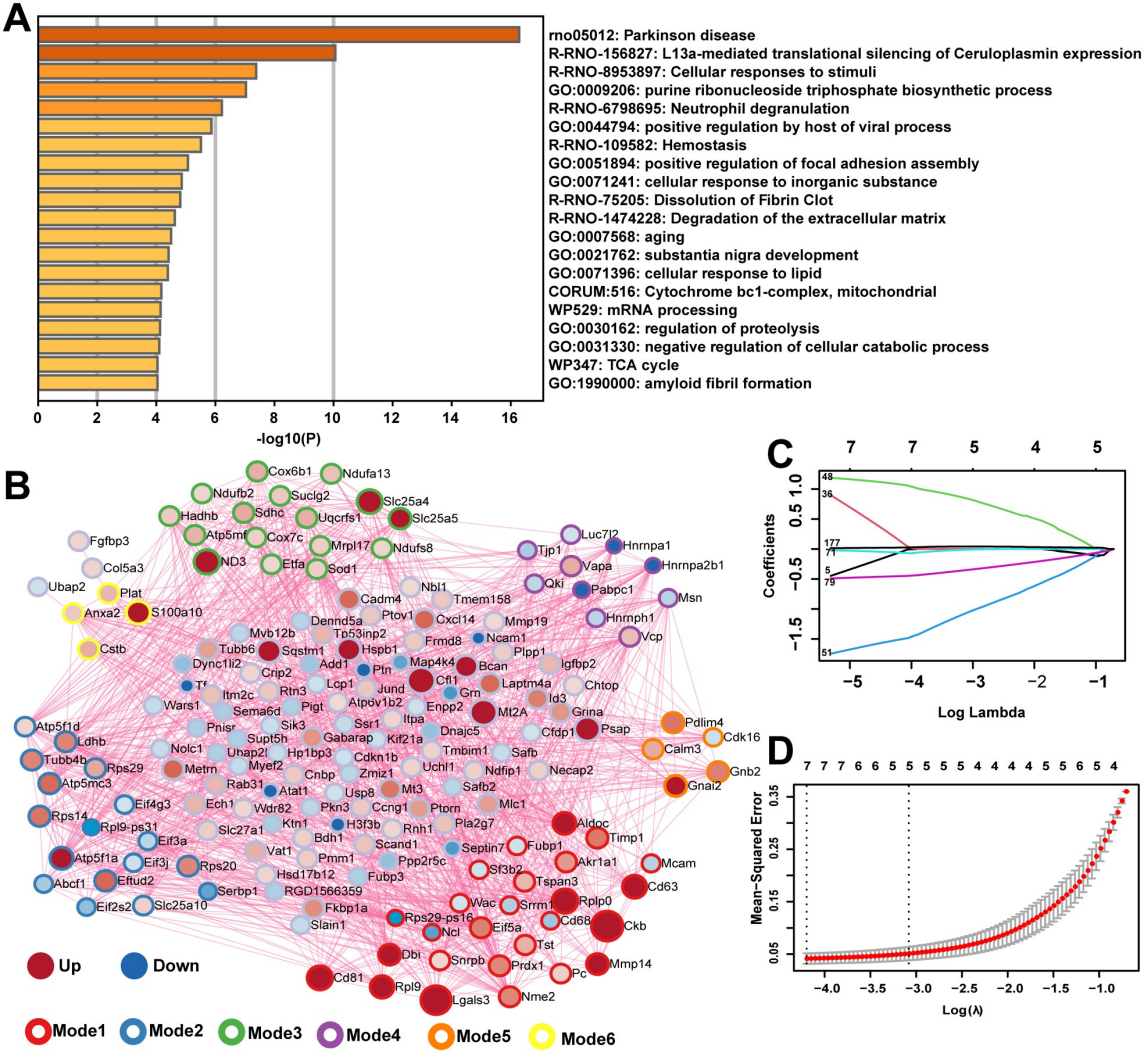
GO enrichment analysis, PPI and LASSO analysis of the candidate genes. **A** Functional enrichment analysis for 195 candidate genes. **B** PPI analysis of 195 candidate genes. An Mode algorithm was applied to the network, and GO enrichment analysis was applied to each Mode network, each Mode network being assigned the color of a stroke. Mode is a collection of nodes with common biological function contributions in PPI. For hub nodes, the size of the node represents the value of Mode. The nodes are red or blue, representing the upregulation or downregulation of the expression level in the glutamate-stimulated group, respectively. The darker the color, the greater the level of differential expression. **C** The locus of change of independent variable coefficient of LASSO analysis. Each curve in the figure represents the change trace of coefficient of each independent variable. The ordinate is the value of the coefficient, the lower abscissa is log (λ), and the upper abscissa is the number of non-zero coefficients in the model at this time. The later the coefficient is compressed to 0, the more important the variable is as the value of lambda changes. **D** Model error diagram of LASSO analysis. On the ordinate is Mean-Squared Error. Cross Validation of LASSO analysis allows that for each λ value, around the mean of the target parameter shown by the red dot, we can get the confidence interval of the target parameter. There are two numerical dashed lines, the line with the lowest error on the left (lambda.min) and the line with the least feature on the right (lambda.1se). PPI, protein–protein interaction; LASSO, least absolute shrinkage and selection operator.

We used METASCAPE [46] to annotate the GO and KEGG pathway for 195 candidate genes, with the result of ranking in the top 20 (Fig 4A). We found that these genes were mainly associated with cell stress response GO Biological Processes, such as *positive regulation by host of viral process*, *positive regulation of focal adhesion assembly*, *cellular response to metal ion*, *cellular response to lipid*, *negative regulation of cellular catabolic process*, and *amyloid fibril formation*; related to *Parkinson disease* KEGG Pathways; related to 5 Reactome gene sets, including *Cellular responses to stimuli*, *Neutrophil degranulation*, *Hemostasis*, *Dissolution of Fibrin Clot*, and *Degradation of the extracellular matrix*. These results suggested that the damaged neurons were stimulated to produce related stress responses. PPI of these candidate genes was analyzed by METASCAPE website (Fig 4B). The larger the Mode-degree value of a pivot node, the larger its shape. Red indicated that the expression level was upregulated after the neurons were damaged by glutamate stimulation, and blue indicated that it was downregulated. Mode is a collection of nodes with common biological function contributions in PPI. The stroke colors for the top six Mode node sets for Log-P values are red, blue, green, purple, orange, and yellow. Different Modes represent different densely connected network nodes. In the main GO enrichment functions of each Mode, the minimum Log-P of Mode3 means that the gene function enrichment analysis at the Mode3 node was more representative (Table 3). Among the results, *Chemical carcinogenesis-reactive oxygen species*, *tricarboxylic citric acid (TCA) cycle and respiratory electron transport*, and *Parkinson disease* were all suggested to be related to the injury and glutamate excitatory stimulation of neurons. In order to screen out the key gene verified in vivo and in vitro, the least absolute shrinkage and selection operator regression analysis (LASSO) was performed on 195 hub nodes (Fig 4C and 4D). The two dotted lines indicated two special λ values, namely, lambda. min and lambda.1se (Fig 4D). Lambda. min is the one that gives the mean of the minimum target parameter of all λ values. Lambda.1se represents the value of a model with good performance and the fewest arguments. Our goal is to select the model of λ corresponding to the variable features as little as possible and the error as small as possible. Because after the λ value reaches a certain size, continuing to increase the number of model-independent variables, that is, reducing the λ value, cannot significantly improve the model performance. Finally, we select the model of λ corresponding to lambda.1se. Finally, we identified six key genes (Anxa2, Ccng1, Hspb1, Lgals3, Timp1, and S100a10) with upregulated expression levels (Table 4).

**Table 3:** GO enrichment description for each mode module. GO enrichment analysis had been applied to each Mode component independently, and the three items with the highest scores calculated by P value are the functional descriptions of the corresponding modes.

Network	Go	Description	LogP
**All**	rno05012	Parkinson disease	-15.1
	rno05208	Chemical carcinogenesis—reactive oxygen species	-13.4
	rno05020	Prion disease	-12.4
**Mode1**	GO:0046364	monosaccharide biosynthetic process	-4.3
	GO:0002181	cytoplasmic translation	-3.7
	R-RNO-6798695	Neutrophil degranulation	-3.6
**Mode2**	R-RNO-72695	Formation of the ternary complex, and subsequently, the 43S complex	-11.1
	R-RNO-156827	L13a-mediated translational silencing of Ceruloplasmin expression	-11.1
	R-RNO-72706	GTP hydrolysis and joining of the 60S ribosomal subunit	-11.1
**Mode3**	rno05208	Chemical carcinogenesis—reactive oxygen species	-17.4
	R-RNO-1428517	The citric acid (TCA) cycle and respiratory electron transport	-17.2
	rno05012	Parkinson disease	-16.7
**Mode4**	GO:0008380	RNA splicing	-8.4
	GO:0006397	mRNA processing	-7.8
	GO:0016071	mRNA metabolic process	-6.9
**Mode5**	R-RNO-111885	Opioid Signalling	-6.4
	rno04713	Circadian entrainment	-5.8
	R-RNO-112314	Neurotransmitter receptors and postsynaptic signal transmission	-5.5
**Mode6**	R-RNO-75205	Dissolution of Fibrin Clot	-9.1
	GO:0030162	regulation of proteolysis	-5.6
	R-RNO-109582	Hemostasis	-4.3

**Table 4:** Coe represents the coefficient value of the gene as a characteristic variable in the results of LASSO. LASSO, least absolute shrinkage and selection operator.

Variable	Coe
(Intercept)	6.034526044
Anxa2	0.003102104
Ccng1	0.0000088029734383845
Hspb1	0.019784375
Lgals3	0.347581692
Timp1	0.002688429
S100a10	0.0000103781562291607
Cyrib	-0.014888626
Msn	-0.1879891
Pkn3	-0.082615714
Tf	-0.002591211

### The key genes expression level comparison and verification

**Fig 5:**
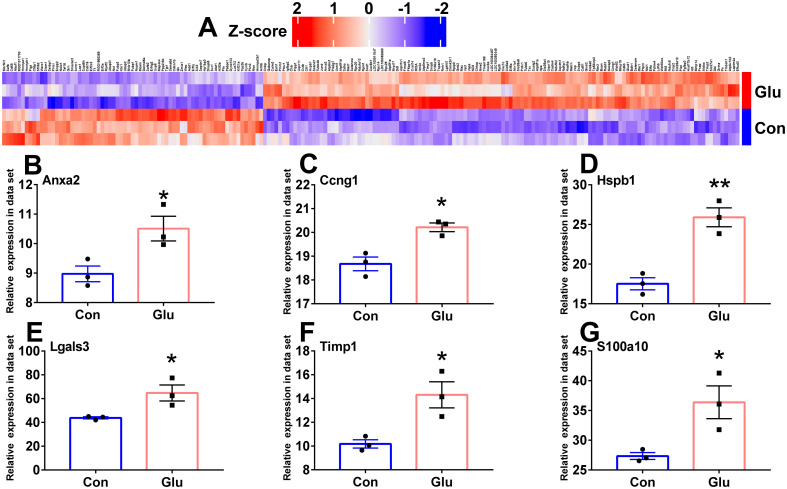
Comparison of hub genes expression levels. **A** Heat map of 195 candidate genes. **B-G** Histogram of relative expression levels of 6 key genes in our sequencing dataset (Unpaired student T-test, n = 3/group). Con, control group; Glu, Glutamate stimulation group. * P < 0.05, ** P < 0.01.

**Fig 6:**
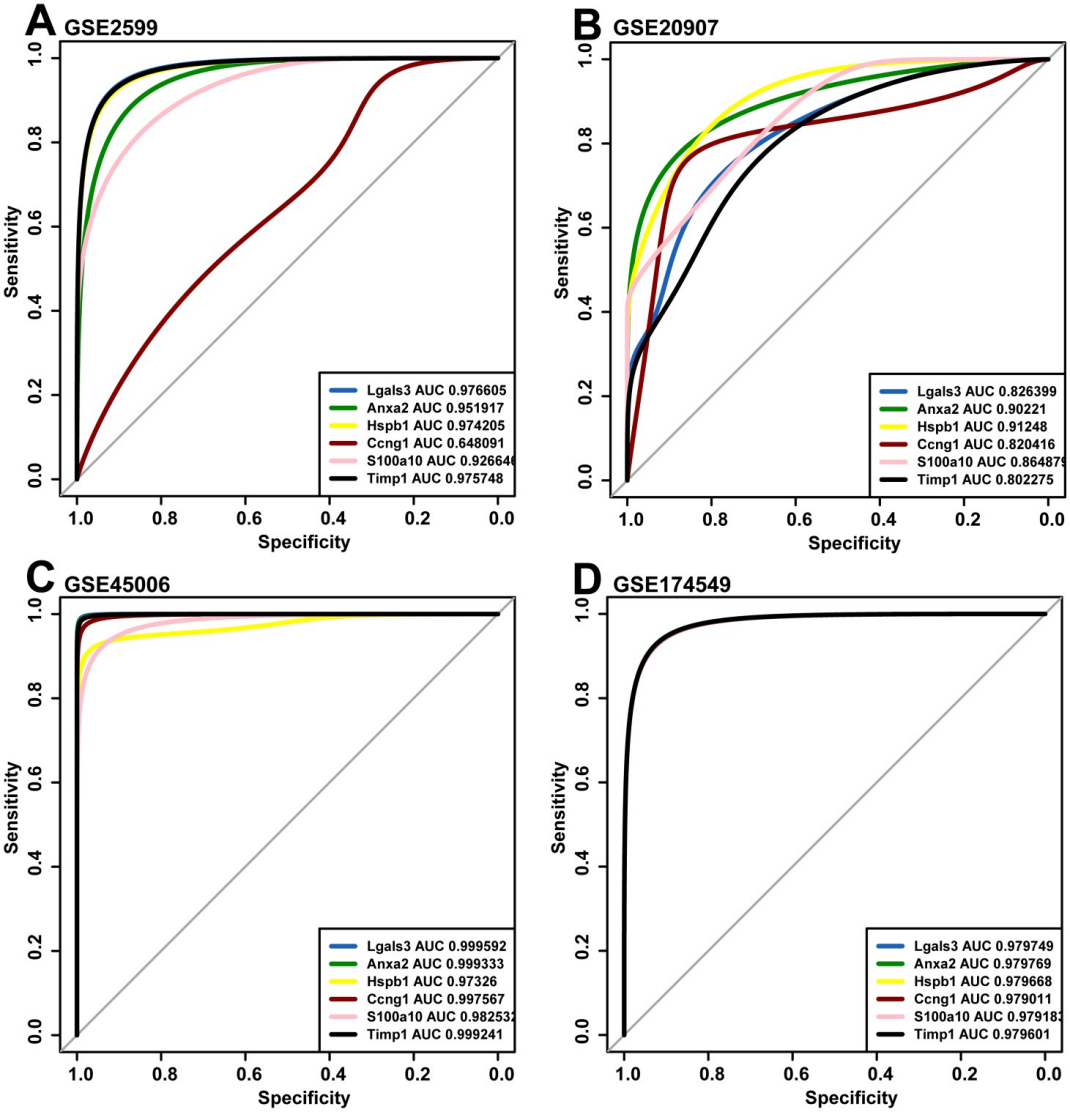
The ROC curve of the corresponding expression levels of the six key genes was verified in GSE2599 (A), GSE20907 (B), GSE45006 (C) and GSE174549 (D). ROC curves show the comprehensive evaluation of the sensitivity and specificity of each key gene in judging whether SCI has occurred in rats in four datasets. AUC is a numerical index of ROC, and the closer the AUC value of the key gene is to 1, the stronger the ability of the gene to judge SCI in rats. ROC, receiver operating characteristic; AUC, area under curve; SCI, spinal cord injury.

**Fig 7:**
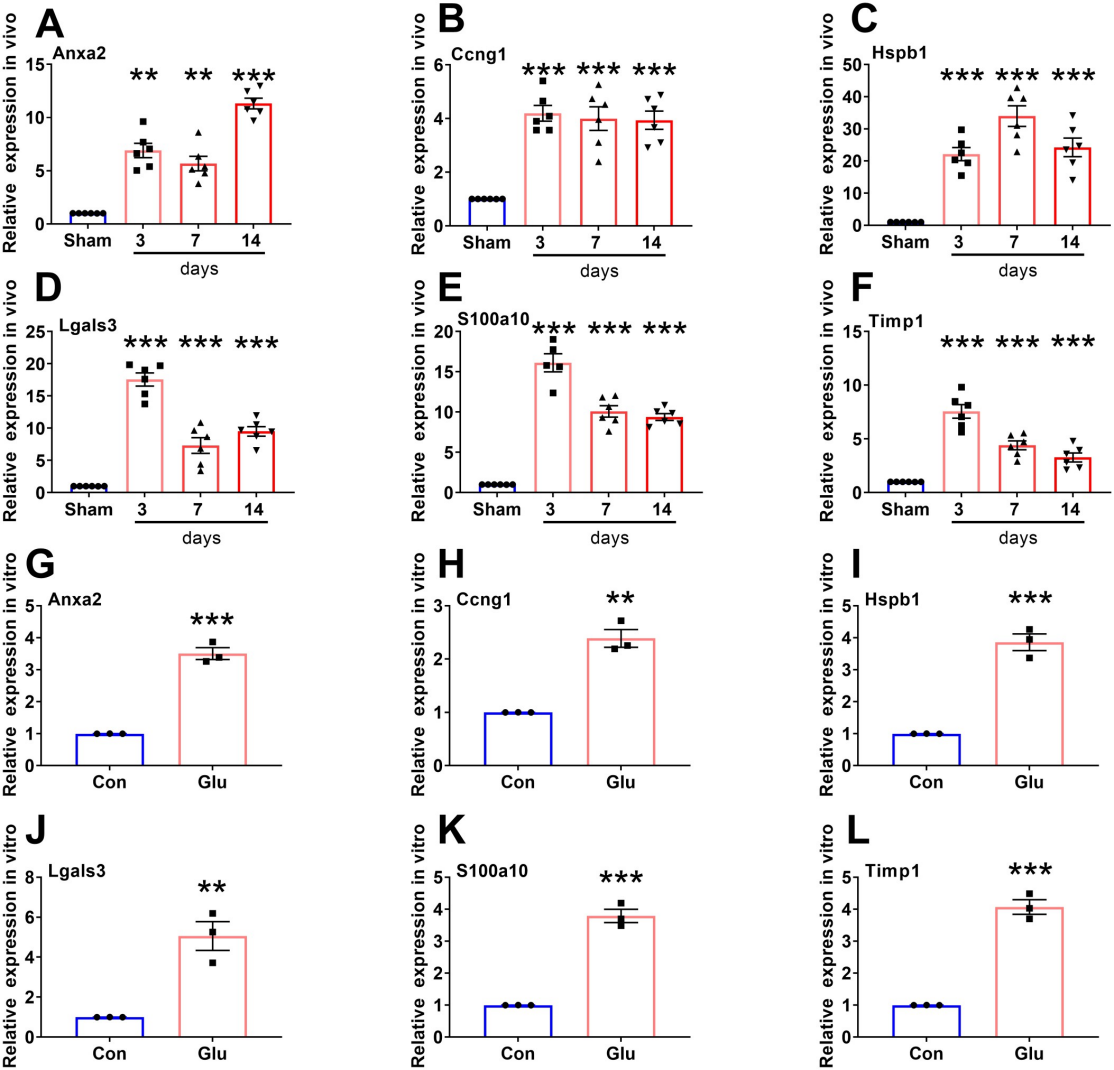
RT-PCR validation in vivo and in vitro. **A-F** The relative expression levels of six key genes in the Sham group and the SCI group rats (One-way ANOVA, n = 6/group). **G-L** The relative expression levels of six key genes in primary spinal cord neuron cell between control group and glutamate stimulation group (Unpaired student T-test, n = 3/group). SCI, spinal cord injury; Con, control group; Glu, Glutamate stimulation group; * P < 0.05, ** P < 0.01, *** P < 0.001.

We compared the expression levels of 195 candidate genes between the control and the glutamate-stimulated group by heatmap (Fig 5A). And the expression levels of six key genes were compared (Fig 5B–5G). In order to validate whether these six key genes can serve as indicators for determining SCI, we downloaded the corresponding expression levels of these genes in GSE2599 [50], GSE20907 [51], GSE45006 [52] and GSE174549 [53]. According to the expression levels of the six key genes, the ROC was drawn to calculate the corresponding AUC area (Fig 6). Except that the AUC value of Ccng1 (AUC = 0.648091) in GSE2599 was relatively small, the AUC values of the six key genes were all greater than 0.8, indicating that the six key genes had good judgment value for SCI. Notably, the relative expression levels of the six key genes in both in vitro and in vivo experiments were consistent with the expression matrix, that is, the expression levels were relatively upregulated and all had statistical differences (Fig 7).

### Correlation analysis between key genes and autophagy

**Fig 8:**
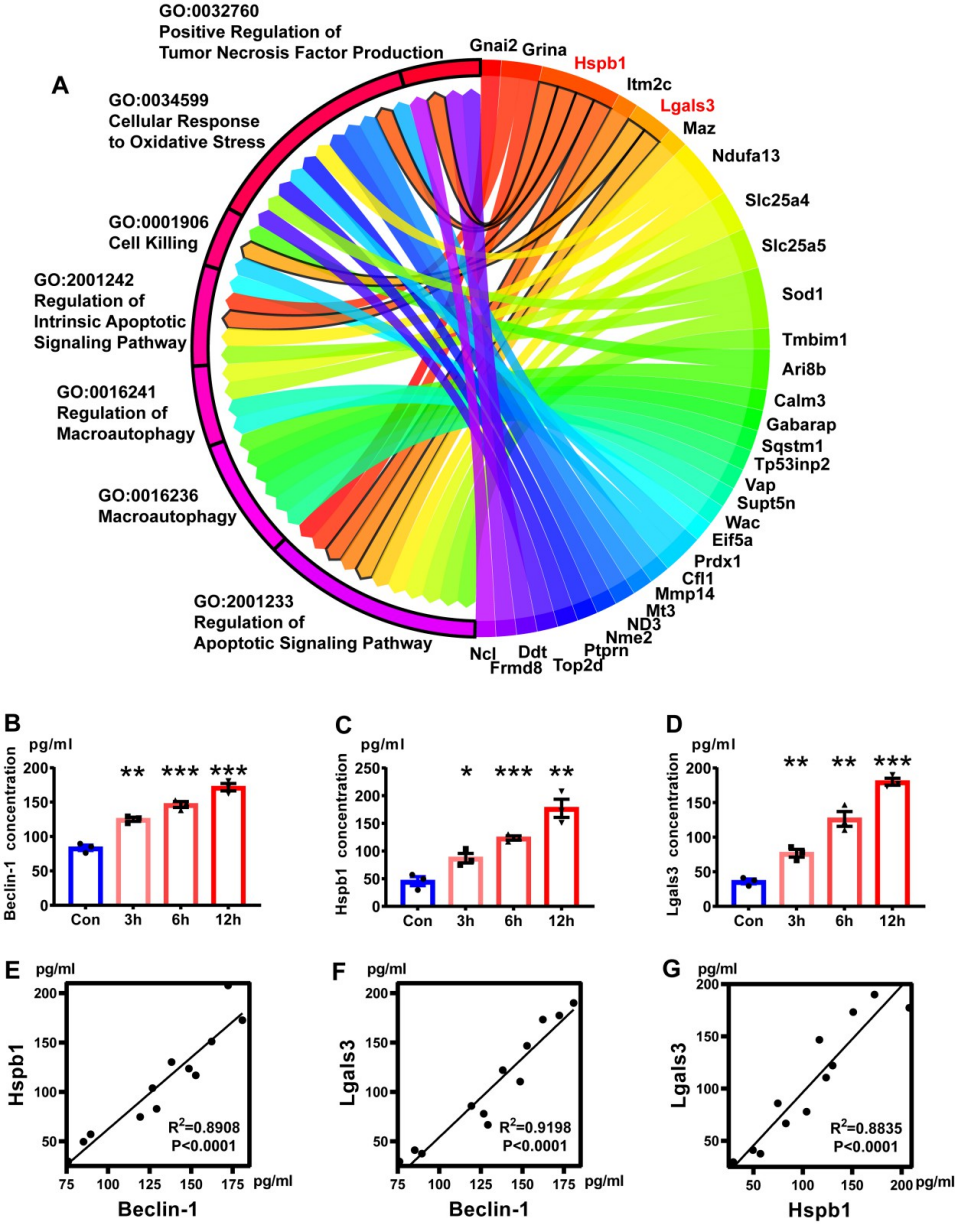
Autophagy correlation analysis. **A** Chord diagram of autophagy. The left side of the picture shows the enrichment results related to autophagy in GSEA, and the right side shows the genes that play a decisive role in each enrichment result. **B-D** The expression levels of Beclin-1, Hspb1 and Lgals3 after glutamate-stimulated primary spinal cord neurons for 3 h, 6 h, and 12 h by ELISA (One-way ANOVA, n = 3/group). **E-G** Correlation analysis of Beclin-1, Hspb1 and Lgals3. GSEA, Gene Set Enrichment Analysis; ELISA, enzyme-linked immunosorbent assay. * P < 0.05, ** P < 0.01, *** P < 0.001.

The results of the GO enrichment analysis of candidate genes were used to explore the correlation between candidate genes and autophagy caused by excitotoxicity. A total of seven GO Biological Processes were autophagy-related, including *Regulation of Apoptotic Signaling Pathway*, *Macroautophagy*, *Regulation of Macroautophagy*, *Regulation of Intrinsic Apoptotic Signaling Pathway*, *Cell Killing*, *Cellular Response to Oxidative Stress*, and *Positive Regulation of Tumor Necrosis Factor Production* (Fig 8A). Chordal Graph suggested that Hspb1 and Lgals3 were strongly correlated with autophagy. Hspb1 plays an important role in these pathways, *Positive Regulation of Tumor Necrosis Factor Production*, *Cellular Response to Oxidative Stress*, *Regulation of Intrinsic Apoptotic Signaling Pathway*, and *Regulation of Apoptotic Signaling Pathway*. Lgals3 plays an important role in *Regulation of Intrinsic Apoptotic Signaling Pathway* and *Regulation of Apoptotic Signaling Pathway*. Additionally, we detected the expression levels of autophagy marker Beclin-1, Hspb1 and Lgals3 by enzyme-linked immunosorbent assay (ELISA) under different glutamate stimulation time, and found that their expression levels increased with the stimulation time (Fig 8B–8D). Through correlation analysis, Beclin-1, Hspb1 and Lgals3 have significant positive correlation with each other (Fig 8E–8G).

## Discussion

In this study, we initially identified a characteristic gene set comprising 195 candidate genes closely associated with excitotoxicity by employing WGCNA, DEG, and RF algorithms. Subsequently, through Metascape and PPI analyses, we inferred that this characteristic gene set is intricately linked to neuronal stress injury. Furthermore, utilizing the LASSO method, we pinpointed six key genes–Anxa2, S100a10, Ccng1, Timp1, Hspb1, and Lgals3. The robust diagnostic significance of these six key genes, as indicated by their Area Under the Curve (AUC) values, underscored their potential utility in distinguishing between four distinct subacute SCI models in rats. Highlightedly, Hspb1 and Lgals3 were identified as pivotal players in regulating neuronal autophagy triggered by excitotoxicity.

In the normative CNS, glutamate clearance by neurons and astrocytes occurs promptly [56]. Post-SCI excitotoxicity primarily stems from extracellular glutamate accumulation, although various specific mechanisms are implicated [3]. Acute phase SCI (< 3 days) is characterized by mechanical injury-induced neuronal and glial cell rupture, accompanied by direct glutamate release [4]. Subacute SCI (3–14 days) witnesses escalated neuronal and glial cell impairment, leading to glutamate receptor (NMDA, AMPA, and kainic acid receptors) saturation and subsequent intracellular calcium influx, culminating in autophagy and apoptosis [3,8,57]. Concomitantly, hindrance in glutamic acid recovery by activated astrocytes via lipid peroxidation exacerbates glutamate accumulation in the SCI milieu [3].

Our study involved the stimulation of primary spinal cord neurons in neonatal rats with high concentrations of glutamate to mimic post-subacute SCI excitotoxicity, followed by transcriptome sequencing analysis. Gene Set Enrichment Analysis (GSEA) results unveiled predominant autophagy, lysosomal stress response, and antioxidant response in spinal cord neurons under glutamate stimulation. Prior research on hippocampal neuron sequencing in mice following varying glutamic acid exposure durations revealed calcium ion dysregulation, organelle stress (lysosomes and endoplasmic reticulum), oxidative stress, and apoptotic pathway activation as primary regulatory mechanisms across excitotoxicity models [58], paralleling our own findings.

To delve deeper into the characterization of key genes implicated in spinal cord neuron excitotoxicity and investigate potential correlations, we undertook an additional analysis of the sequencing data using a machine learning algorithm. In comparison to conventional signal processing methods, machine learning algorithms offer numerous advantages, including enhanced computational efficiency and the capacity to derive optimal model solutions using sample data [59]. Notably, well-established machine learning algorithms such as WGCNA and RF have been extensively applied in biomedical research. WGCNA primarily focuses on specific phenotypes and co-expression modules, which represent sets of genes that are concurrently upregulated or downregulated under varying conditions [60,61]. By leveraging weighted correlations to gauge gene connectivity, WGCNA captures nonlinear relationships and intricate network structures between genes, thus enhancing the precision and stability of differential expression analyses [60,61]. In contrast, Random Forest assesses variable importance during categorization decisions [62,63], while the LASSO method tailors model attributes to select optimal solutions [64]. Integrating diverse machine learning methodologies allows for the amalgamation of each algorithm’s strengths, leading to enhanced prediction accuracy, reduced potential for bias and overfitting specific datasets, and facilitating a more comprehensive understanding of underlying biological characteristics for researchers.

Anxa2, a Ca^2+^-dependent phospholipid binding protein, plays a pivotal role in excitotoxicity-induced intracellular Ca^2+^ elevation and subsequent cell demise [65]. Additionally, Anxa2 functions as an RNA binding protein, with phosphorylated Anxa2 monomers in the cytoplasm binding to the 3’ untranslated regions of c-Myc mRNA. This interaction results in the perinuclear localization of c-Myc and contributes to cell processes including proliferation, differentiation, and apoptosis [66]. Anxa2 is extensively involved in diverse cellular activities such as apoptosis, migration, membrane repair, and inflammatory responses, all of which significantly influence disease prognosis and severity [67,68]. Previous research has established the direct involvement of Anxa2 in enhancing glutamate-induced cell death in the retinal ganglion cell line RGC-5 [69]. Ccng1, a member of the cyclin family, participates in growth regulation and is associated with G2/M phase arrest following DNA damage [70]. Studies have demonstrated Ccng1’s ability to promote apoptosis in MPC5 cells and induce selective autophagic degradation in hepatocellular carcinoma by impeding G cell cycle progression and inhibiting DNA synthesis [71]. S100a10, also known as p11 or the Anxa2 light chain, forms a complex with Anxa2, creating symmetrical connections between opposing membrane surfaces that induce vesicle aggregation [72]. Both S100a10 and Anxa2 play crucial roles in IFN-γ-induced autophagy. S100a10 facilitates IFN-γ-induced autophagy by modulating the localization of the autophagy initiator ULK1 at the ER-mitochondria contact site [73]. Timp1, a natural inhibitor of matrix metalloproteinases (MMP), plays a role in regulating cell differentiation, migration, and cell death [74]. Research has shown that amiodarone-induced secretory autophagy enhances Timp1 secretion, inhibiting lung cancer cell movement both in vivo and in vitro [75]. In severe traumatic brain injury (sTBI), Timp1, acting as an inflammatory cytokine, has been closely linked to disease severity [76]. It should be pointed out that the mechanism of four key genes in excitotoxicity and autophagy is rarely reported.

It is noteworthy that Hspb1 and Lgals3 are significantly enriched in the autophagy gene set of neurons. With prolonged exposure to excitotoxicity, the expression levels of autophagy markers Beclin-1, Hspb1, and Lgals3 in neurons continue to increase. Moreover, Beclin-1 was found to be positively correlated with both Hspb1 and Lgals3, suggesting that Hspb1 and Lgals3 play a crucial role in the autophagy mechanism induced by excitotoxicity. It has been suggested that Hspb1 may be involved in the antioxidant defense of astrocytes, protecting them from excitotoxicity [77,78]. Overexpression of Hspb1 has been shown to ameliorate symptoms of hypoxic-ischemic brain damage in the hippocampus of rats and exert neuroprotective effects [79]. However, the specific mechanism of Hspb1 in neurons remains to be elucidated and requires further investigation. Furthermore, although many studies have demonstrated the important role of Lgals3 in autophagy [80,81], there are conflicting reports on its relationship with autophagy flux. Some studies have shown that Lgals3 can promote autophagy, while others have found that it can inhibit autophagy [82–84]. Recent research has revealed that Lgals3 secreted by injured neurons can activate and reprogram microglia in the spinal dorsal horn, leading to neuropathic pain [85]. Despite its association with neuroinflammation [86], Lgals3 has been overlooked in the study of neuronal autophagy.

Furthermore, a controversy exists regarding the relationship between excitotoxicity and autophagy [87]. In SHSY5Y neuroblastoma cells [88] and PC12 cells [89], low concentrations of physiological glutamic acid stimulation have been shown to induce autophagy, thereby promoting cell survival. Conversely, high levels of glutamate can lead to neuronal apoptosis, autophagic cell death, or other forms of cell death [90,91]. In the cortex, neurons typically exhibit simultaneous characteristics of autophagy and apoptosis following injury caused by excitotoxicity. Instances of purely autophagic (Type II) cell death or purely apoptotic (Type I) cell death have not been reported [92]. Elevated levels of both autophagy and apoptosis have been observed in the penumbra region following cerebral ischemia, indicating an interaction between these two modes of neuronal death [93,94]. However, in the hippocampus, neuronal death predominantly manifests as either pure apoptosis or pure autophagy [95]. It is worth noting that various cell death mechanisms, such as autophagy, apoptosis, necroptosis, and necrosis, have been extensively studied and found to be involved in the neuronal death processes following SCI [4]. Nevertheless, further investigation is needed to elucidate the impact of injury duration and severity on the neuronal death mechanisms.

In fact, the interaction between multiple cells and molecular crosstalk within the pathological microenvironment has been a key focus of SCI research. The time-dependent transition of astrocytes and reactive microglia following SCI has been identified as a critical aspect for SCI treatment [96]. The pro-inflammatory milieu following central nervous system injury modulates microglia, astrocytes, and infiltrating macrophages, influencing changes in neuronal autophagy levels. Glial cells are capable of releasing various extracellular signaling molecules, such as TNF-α and IL-1β, forming a glial network through intimate interactions that impact the regulation of neuronal autophagy. It has been suggested that decreasing glial cell proliferation and neuronal autophagy can mitigate the neuroinflammatory response [97]. In addition, neurons can transmit autophagic signals to astrocytes, preventing the release of damaged mitochondria and restricting neuroinflammation, as seen in Parkinson’s disease [98]. Furthermore, glial cells engage in the removal of accumulated protein aggregates, such as abnormal Tau protein or α-amyloid β-protein, via phagocytosis, coordinating with neuronal autophagy processes. Dysfunction in autophagy plays a pivotal role in neurodegenerative diseases [99]. The impact of glial-neuron interactions within the pathological microenvironment on neuronal autophagy can be both beneficial and detrimental [23,96,99].

In summary, our research highlights the significant roles of Anxa2, S100a10, Ccng1, Timp1, Hspb1, and Lgals3 in the excitotoxicity mechanism affecting neurons post-SCI. Notably, Hspb1 and Lgals3 may be implicated in the regulation of neuronal autophagy triggered by excitotoxicity. Our findings offer compelling evidence regarding the neuropathic mechanisms involving excitotoxicity of these key genes post-SCI. A comprehensive understanding of the mechanisms underlying excitotoxicity and autophagy-triggered cell death resulting from SCI necessitates thorough investigation of various factors, including cell types, time points, molecular mechanisms, and tissue environments. Our study suggests that Hspb1 and Lgals3 may represent novel targets for SCI treatment, enhancing comprehension of excitotoxicity and autophagy processes in spinal cord neurons and proposing fresh perspectives for SCI therapy.

## Conclusions

Our study offers a biological functional characterization of neuronal excitotoxicity induced by high concentrations of glutamate. We observed up-regulation of Anxa2, S100a10, Ccng1, Timp1, Hspb1, and Lgals3 in neurons following stimulation with high levels of glutamate, underscoring their potential significance in the diagnosis of SCI. Furthermore, Hspb1 and Lgals3 are closely associated with neuronal autophagy triggered by excitotoxicity. This insight is crucial for comprehending the impact of excitotoxicity on neurons post-SCI and its implications for the underlying mechanisms of neuronal demise.

## Supporting information

S1 Fig Autophagy correlation analysis. Determination of optimal glutamate concentration and duration using CCK8 assay.
(TIF)

S1 Table Biological process analysis between control group and glutamate-stimulated group of spinal cord neurons. (XLSX)

S2 Table Cellular component analysis between control group and glutamate-stimulated group of spinal cord neurons. (XLSX)

S3 Table Molecular function analysis between control group and glutamate-stimulated group of spinal cord neurons. (XLSX)

S4 Table KEGG analysis between control group and glutamate-stimulated group of spinal cord neurons. (XLSX)

S5 Table Reactome analysis between control group and glutamate-stimulated group of spinal cord neurons. (XLSX)
