# Supplementary figures and images for: Hspb1 and Lgals3 in spinal neurons are closely associated with autophagy following excitotoxicity based on machine learning algorithms
Source: PLoS One. 2024 May 10;19(5):e0303235. doi: 10.1371/journal.pone.0303235 (PMC11086895; doi:10.1371/journal.pone.0303235)

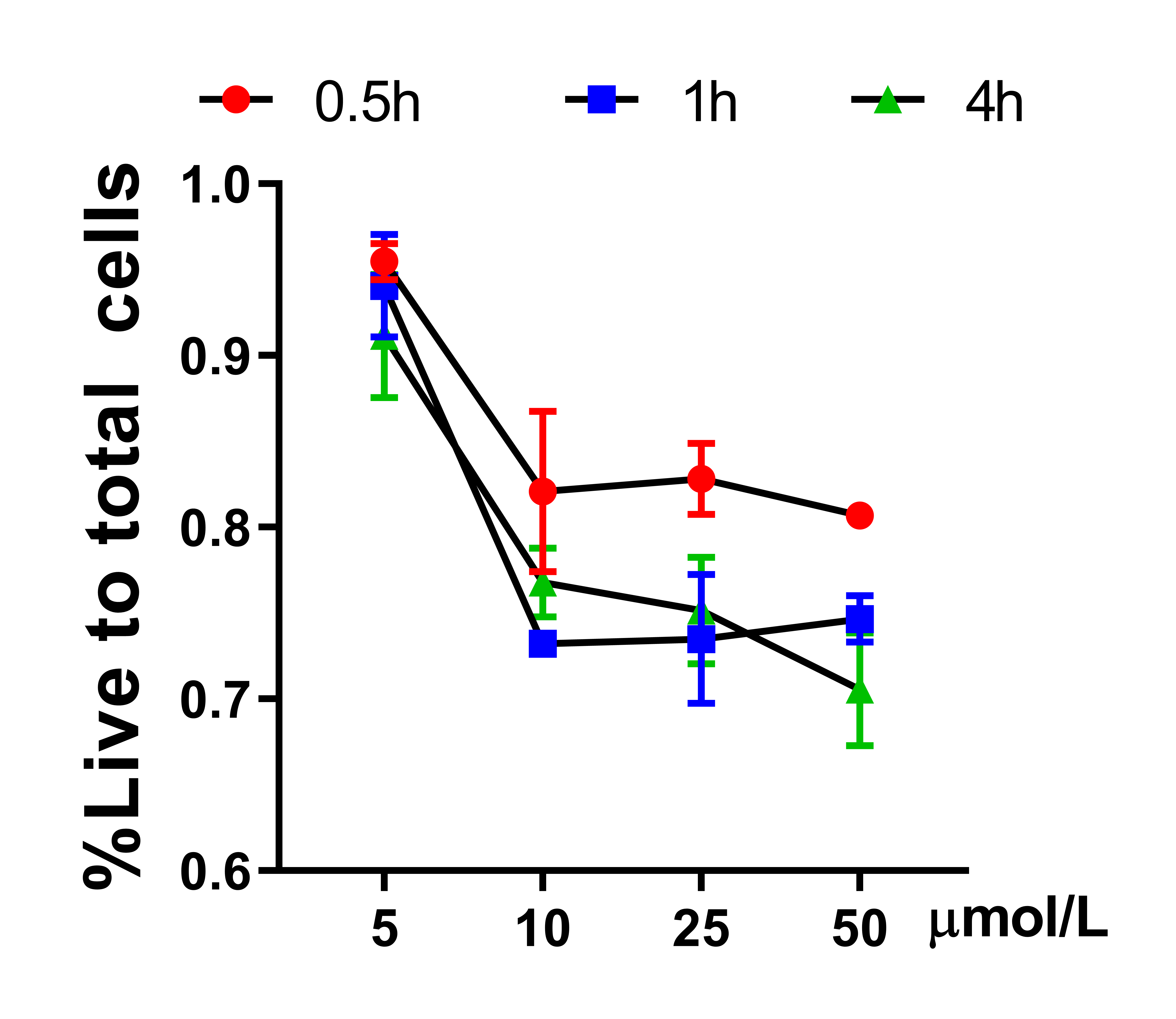

Supplement: S1 Fig — Determination of optimal glutamate concentration and duration using CCK8 assay. (TIF) [file pone.0303235.s001.tif]
